# Freeze concentrated apple juice maintains its flavor

**DOI:** 10.1038/s41598-021-92274-0

**Published:** 2021-06-16

**Authors:** Tsuyoshi Yoda, Hiroshi Miyaki, Tomoaki Saito

**Affiliations:** 1 Aomori Prefectural Industrial Technology Research Center, Hachinohe Industrial Research Institute, 1-4-43 Kita-inter-kogyodanchi, Hachinohe City, Aomori 039-2245 Japan; 2 Aomori Prefectural Industrial Technology Research Center, Hirosaki Industrial Research Institute, 1-1-8 Ougi-machi, Hirosaki City, Aomori 036-8104 Japan; 3 The United Graduate School of Agricultural Sciences, Iwate University, 3-18-8, Ueda, Morioka 020-8550 Japan

**Keywords:** Chemistry, Engineering, Materials science

## Abstract

Concentrated juices are sources of alcoholic drinks. Juice concentration may be achieved using different methods, such as freezing or heating. High temperatures in the process of juice concentration damage heat-sensitive components, such as aromatic compounds. Although the freezing process of juice concentration has been studied, analyses have been inadequate, particularly in addressing flavors. Therefore, we investigated the characteristics of freezing and heating during apple juice concentration in the context of flavor. We found that a total of 97 compounds were found in fresh juice, and freeze-concentrated juice retained 57 of these compounds. Interestingly, freezing led to the generation of 37 flavor compounds. Furthermore, people had difficultly differentiating between intact and frozen concentrated juice. The ratios were almost same between those who correctly identified (28%) and those who incorrectly identified fresh and reconstituted freeze-concentrated juice (25%). We discuss the mechanisms of flavor generation on freezing concentration with regard to the increases in enzymatic activity or other causes. Our study showed that the methods of juice concentration that utilize freezing retain flavor better. These data will benefit juice concentration processes of apples and other fruits in the future.

## Introduction

Concentrated juices are sources of alcoholic drinks in the fermentation process^1,2^. Juice production is one of the most important applications of the fruit-processing industry^3^. During juice production, different concentration processes may be employed, such as concentration by freezing or heating.

Juices are usually concentrated by heating evaporation; however, the high temperatures used in these processes are known to damage heat-sensitive components and reduce the quality of the juice^4^. Therefore, membrane techniques have been developed for the concentration step in industrial applications of fruit juice^5,6^.

Juice concentration via freezing has been studied widely^2^. This technique employs methods to remove water as ice crystals by cooling the fluid to be concentrated to temperatures below the freezing point of the juice^7,8^. The greatest advantage of concentrating juice by freezing is that, unlike the heat concentration method, freezing retains flavors^9^. It has been reported that freeze-concentrated juice is not different in flavor from the original juice, according to the panelists in an organoleptic test^10^. However, these studies are highly dependent on the senses of individual subjects and lack objective chemical analyses of flavors.

The quality of many types of fruit juices concentrated by various methods has been investigated by gas chromatography–mass spectrometry (GC–MS)^2,11–13^. Human tasting is also used for quality control of apple juice^14^, and its concentrated form is often used to study its quality^15,16^. Recently, both chemical analysis evaluation and sensing examination of cryoconcentrated juice by humans have been reported^17,18^. The cryoconcentration process does not significantly affect the sensory evaluation results^18^.

In this study, we investigated the characteristics of freezing and heating methods for concentrating the sugar and flavor components of apple juice. Furthermore, we conducted sensory evaluations to distinguish between fresh and concentrated juices.

## Materials and methods

### Samples and reagents

Apple juice made from Jonagold apples was the kind gift of a juice maker in the Aomori prefecture. We used juice without filtration. Sucrose was obtained by Kanto Chemical Co., Inc. (Tokyo, Japan).

### Freezing concentration

**Figure 1:**
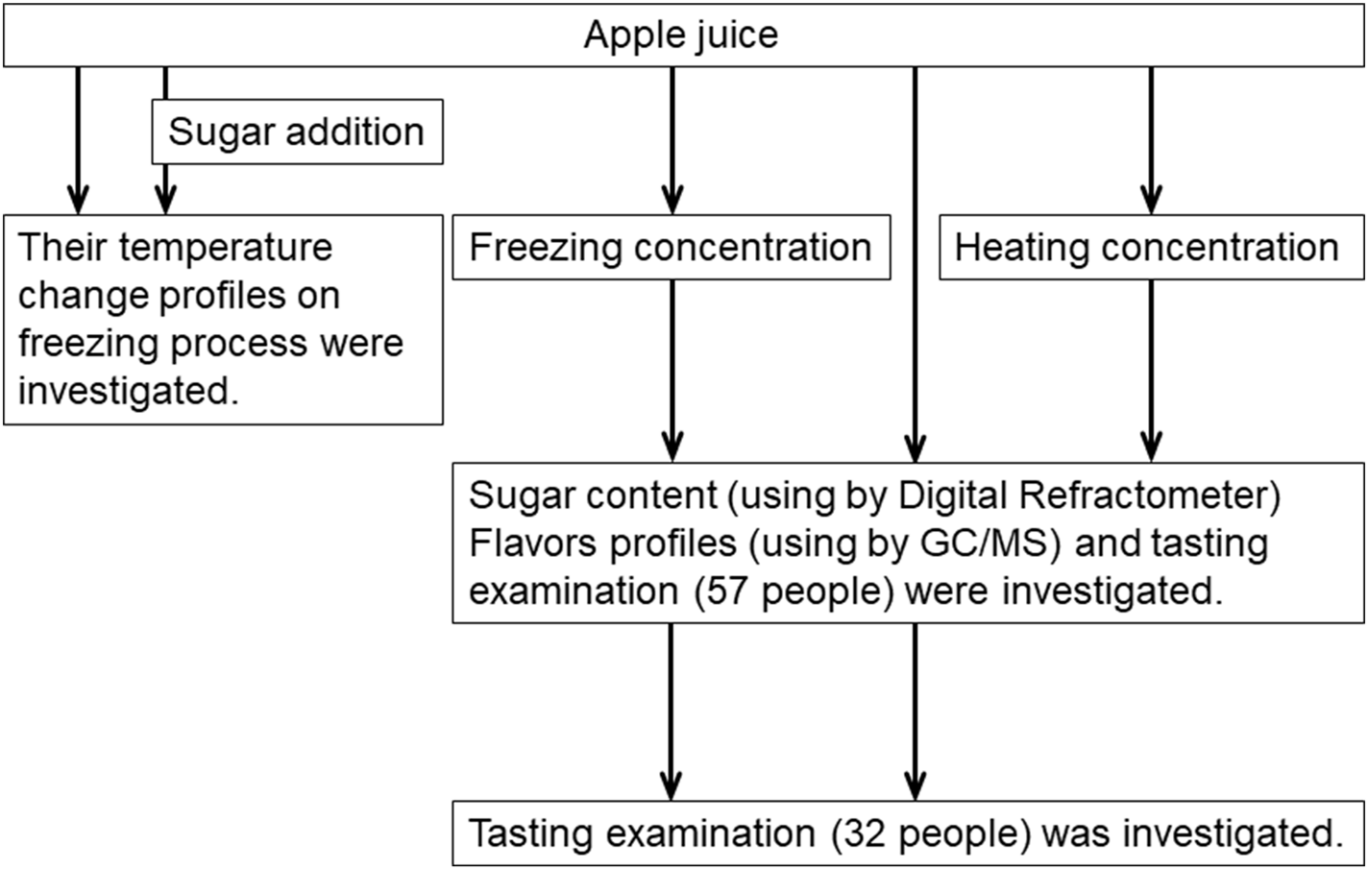
Process diagram of the study.

Apple juice (1 L) was subjected to freezing at − 9.5 °C for several hours using the TouminS-220 W (Technican ltd, Tokyo, Japan), which allowed us to profile the temperature because it did not freeze above a previously set temperature of − 9.0 °C (we initially started at − 5.0 °C and reduced the temperature in 0.5 °C increments). Temperatures were recorded with a temperature logger (TM-947SD J; Lutron Electronics Co., Inc., Tokyo Japan). Ice crystals derived from the apple juice were then obtained and allowed to melt naturally at room temperature. We obtained melted concentrated juice and carefully poured it into a separate beaker. Freezing-point depression was applied to yield a highly concentrated sample. Once approximately 50% of the ice crystals had melted, the liquid that was generated was separated as freeze-concentrated juice. Concentration by freezing can also be performed using a common freezer (Hoshizaki Corporation, Aichi, Japan) overnight. These experiments were performed at least thrice. Error bars denote standard error of the mean (SEM). The process of the study is summarized in Fig. 1.

### Measurement of sugar content

The sugar content in brix (1 g sucrose per 100 g liquid) was measured using the PR-201a Digital Refractometer (Atago Co. Ltd., Tokyo, Japan), which was calibrated to read “0” when only ultrapure water was present.

### Heat concentration

Potted juice (1 L) in a beaker was placed in the heater (SR-550, Advantec Co., Ltd., Tokyo, Japan). The apple juice was heated to boiling (around 100 °C) for 2–3 h. Heating was stopped when the volume had reduced to approximately 50% of the original amount. Experiments were carried out at least three times. Error bars reflect SEM.

### GC–MS analysis

Juice samples were analyzed by GC–MS (Agilent HP5890 SeriesII/5972A MS; Agilent Technologies, Santa Clara, CA, USA)^15,16^ using stir bar sorptive extraction (SBSE) methods^19^. A three-octanal internal standard and 1 g sodium chloride was added to each sample. Measurement solutions absorbed polydimethylsiloxane twisters^20^ with a stirrer at 800 rpm for 1–1.5 h at room temperature (22.5 °C). The analytes were separated on an HP-INNOWAX column (60 m × 0.25 mm × 0.25 μm) from Agilent Technologies using helium as the carrier gas at a constant flow of 1.0 mL/min. Thermal desorption of twister stir bars was performed by ramping thermal desorption unit from 40 to 240 °C (15 min) at a rate of 3 °C/min in the splitless mode under helium flow of 1.0 mL/min. The scanning condition was 30–350 m/z. The measurement protocol was similar to that of official flavor analysis methods employed by our institute^20^. Detected compounds and flavors were analyzed using software provided with the hardware.

### Tasting examination

We used water to dilute heat- or freeze-concentrated juice (concentrated per methods above) to the same brix as that of fresh juice. Randomized volunteers attending a festival event introducing our institute were asked to blindly taste juice samples and match each with its appropriate source (fresh, freeze-concentrated, or heat-concentrated) as a triangle test. A total of 57 volunteers aged 3 to over 70 years participated in the study and included 19 men and 38 women. Another group of participants was asked to choose only between fresh juice and reconstituted freeze-concentrated juice using the same methods as above as a two-point test. This examination included 32 participants aged 3–60 years—18 men and 14 women. Given the wide age range of our volunteers and the inclusion of men and women, our results did not discriminate by age or sex. The data were analyzed and results were considered statistically significant if the *p* value was ≤ 5%. All experiments were conducted in accordance with the relevant guidelines and regulations and were conducted under the approval of both the general manager’s meeting and the open executive committee of Aomori Industrial Technology Center Hirosaki Institute and public relations committee of the Aomori Industrial Technology Center. Our institute does not have a specific committee for research ethics. The ethical concerns of the research were authorized by the director of our research institute after the general manager’s meeting. The experiments were conducted at an open executive event as well as at a center festival. Therefore, the research was conducted after the approval of the open executive committee of Aomori Industrial Technology Center Hirosaki Institute and the public relations committee of the Aomori Industrial Technology Center.

All the participants provided written informed consent, which was obtained both in writing and verbally after providing adequate explanation of the study. In addition, in cases of younger children who participated with their parents, approval from parents was considered as informed consent.

### Statistical analysis

Our experiment was performed at least thrice. One-way ANOVA in Excel was used to evaluate statistical significance of the differences. Data are presented as the mean ± standard error.

## Results and discussion

### Temperature change for freezing juice

The objective of adding sugar was to confirm that small differences in sugar content do not significantly change the freezing temperature. The sugar content of the fresh juice used in this study was 11.8%. Sugar was added to the juice to reach 13.9% because the sugar content of apple juice fluctuates depending on seasonal changes and the type of apples^21^. Kajikawa reported that growth conditions and cultivation techniques are other factors that affect the sugar content of apple juice^21^. For example, in our previous study, we used apple juice with 14.0% sugar content^22^, whereas Orellana-Palma et al. used apple juice with 13.9% sugar content^18^. Moreover, one group that used the same apple juice found that the sugar content was 10.0% and 10.5% when measured at different timepoints^23,24^. Owing to such differences in the type of apple juice, we conducted our study using largely similar conditions as those of this previous report^18,22–24^.

**Figure 2:**
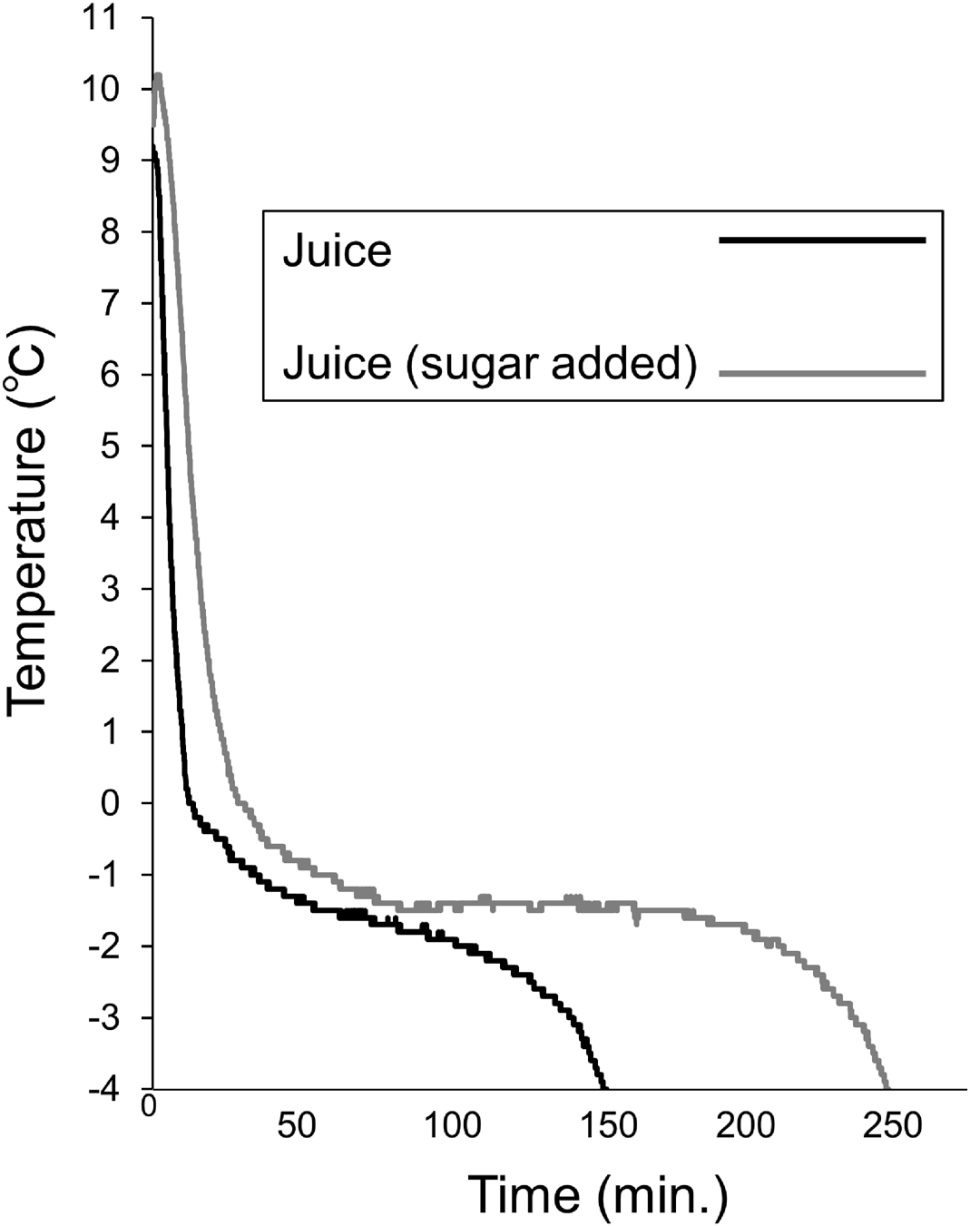
Temperature change for freezing of juice. Temperature changes were measured for normal apple juice (black) and sugar added juice (gray).

Samples of fresh apple juice with or without added sucrose were placed in a liquid freezer to freeze. Temperatures were recorded (Fig. 2) to determine freezing points, indicated by stable temperatures during the phase change from liquid to solid. We revealed that small differences in sugar content did not significantly change the freezing process. Therefore, we regarded the independent value as sugar content and the dependent value as the freezing point. The freezing point of both fresh juice and fresh juice with sugar added was − 1.5 °C. Therefore, the freezing point of the apple juice was not affected by the addition of sucrose, suggesting that changes in sucrose concentration ± 2% need not be considered for freezing temperature adjustments. In a study of freezing of different fruit juices^25^, Safiei et al. reported temperature changes very similar to those observed here. According to their graph, the freezing point of juice was approximately − 1.5 °C, even when they used juice with 16% sugar content. Our findings are also consistent with studies that found that the freezing point was  − 1.6 °C (similar to our findings) for juice with a sugar content of 14% calculated their reported equation^23^ and the relationship between freezing point and suspension conditions in apple juice^24^. We next concentrated the apple juice by heating or freezing.

### Properties of concentrated juice

**Figure 3:**
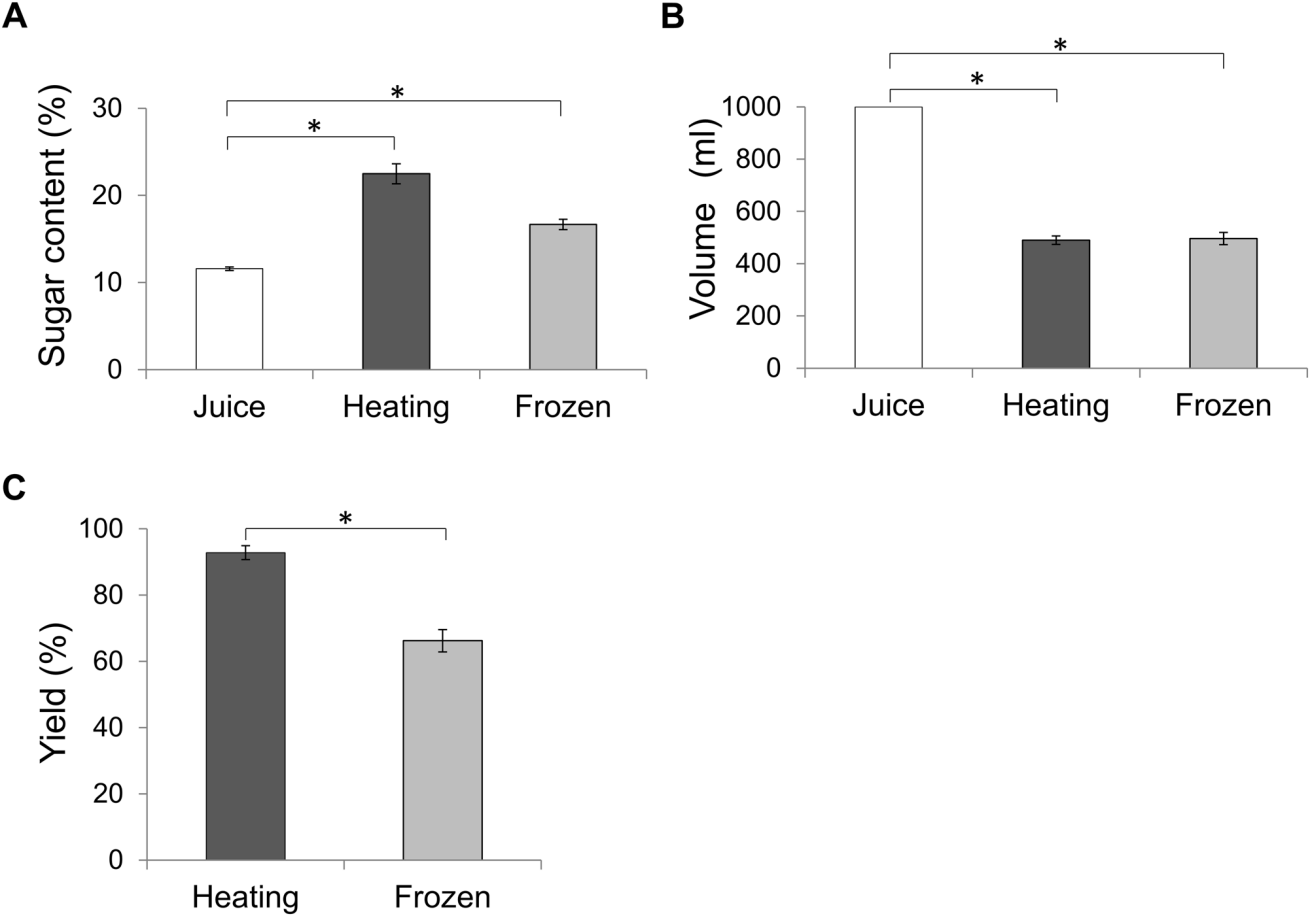
Properties of concentrated juice. Sugar content, volume, and yield are shown in (**A**–**C**), respectively. White: untreated juice; dark gray: heat-concentrated juice; and light gray: freeze-concentrated juice. Sugar content, volume, and yield differed significantly between samples (*P* < 0.05).

Heating and freezing concentration were performed. The color of the apple juice after heat concentration changed from yellow to red, whereas the fresh and frozen juices were very similar in appearance. The actual sugar content, volume, and yield are shown in Fig. 3. Yields were calculated by following equation:

$${\text{Yield~}}\,\left( \% \right) = 100 \times \frac{{final\,sugar\,content~ \times final\,volume}}{{initial\,sugar\,content~ \times ~initial\,volume}},$$

where yield refers to sugar yield. The fresh juice contained approximately 12% sugar content. Heat concentrating increased the sugar content to 22%, whereas freeze concentrating increased the sugar content to 17.0%. Both concentration methods were terminated when the initial volume of 1000 mL reached approximately 500 mL (approximately 50.0%). The calculated yields are shown in Fig. 3C. Heating resulted in a more favorable amount of concentrate than freezing, which rendered a yield of 92.8% versus 66.2%. As ice crystals were thrown out immediately as unnecessary materials in the present study, we did not investigate the contents of the extract. However, the sugar content may be approximately 4.3%, for which the value was calculated from our previous study that investigated the sugar concentration for melted each 10 percent fractions for apple juice^22^. Previously reported ranges of sugar content for freezing concentration were 13–20% in orange juice^26^, 8–60% in watermelon juice^27^, and 20–55%^18,23,24^ in apple juice. Therefore, our concentration method might be further improved to obtain a content of sugar. Heat- and freeze-concentrated juices produced different smells; therefore, flavors analyses were carried out using fresh juice as a reference.

### Degree of flavor retention

**Figure 4:**
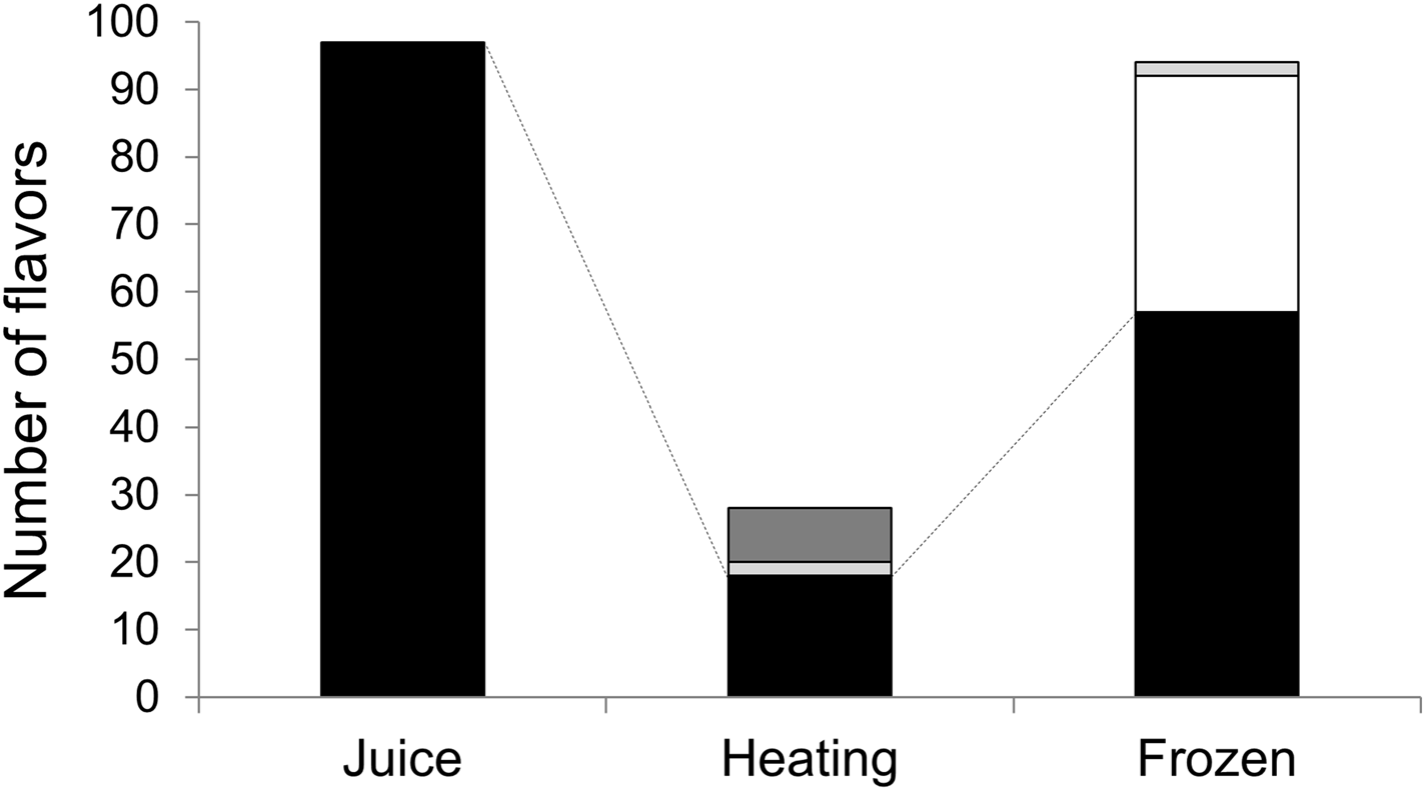
Numbers of flavors in each juice sample. Juice content (black), concentrated generated (light gray), frozen generated (white), and heating generated (dark gray).

Next, we investigated the content of all compounds present in fresh, heat-concentrated, and freeze-concentrated apple juice using the GC–MS (Fig. 4). A total of 97 compounds were found in the fresh juice, but the heat- and freeze-concentrated juices retained 18 and 57 of these exact compounds, respectively. In addition, 8 and 35 new compounds were generated during the heat- and freeze-concentration methods, respectively. Only the compound types and numbers were characterized, and quantities were not measured. More than half of the original flavors in fresh juice were retained during freezing, although not during heating. Interestingly, many flavor compounds were generated during the freezing process, and similar phenomena have been reported previously^18,28^. It has been shown that some apple juice flavors are lost during the heat concentration process^2^. These reports claim that freeze concentrating has advantages over heat concentrating to keep flavors, although 20% of flavors may be lost, and our present results agree with these previous reports^2,29^. New flavors have been detected in freeze-concentrated juice in previous studies^18,28^. The studies suggested that one of the mechanisms for the easy detection of new flavors is to detect the high sugar contents of the freeze-concentrated juice in the headspace^30^. The method we used for flavor detection was not the headspace method because we used the SBSE method. Although SBSE methods might be high concentration of sugar make more easily detection, there may be other mechanisms involved, such as flavors being generated during the freezing process. Therefore, possible mechanisms will be discussed in more detail in subsequent sections.

In a previous study, researchers noted that the flavor components in apple juice decreased after heat treatment^31^, which is in agreement with our results.

**Figure 5:**
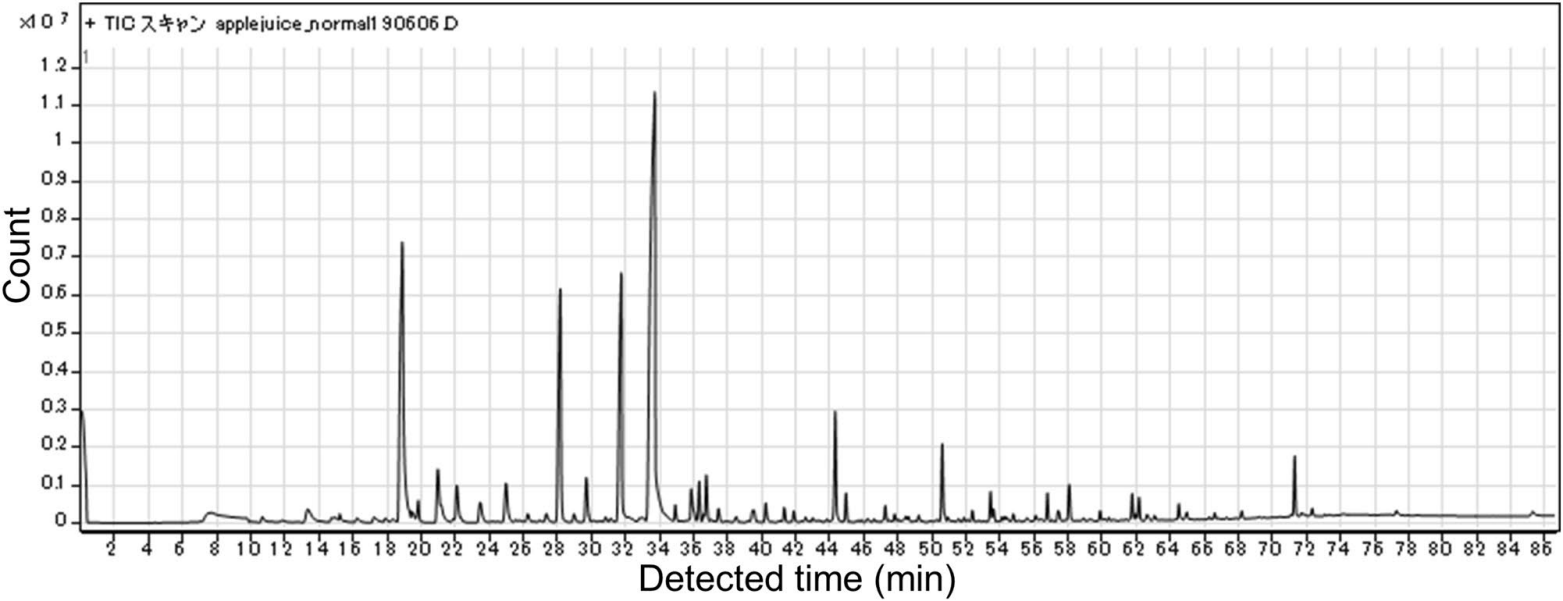
Chromatogram of fresh apple juice.

**Figure 6:**
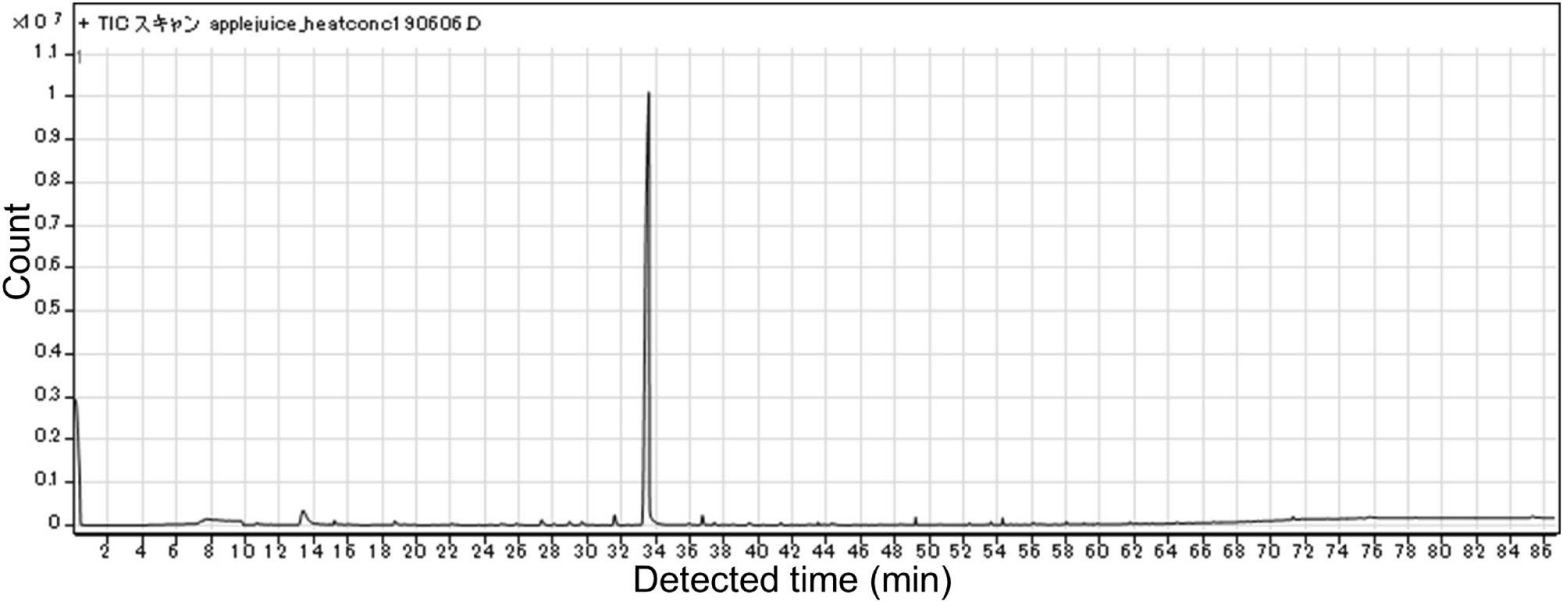
Chromatogram of apple juice concentrated by heating.

**Figure 7:**
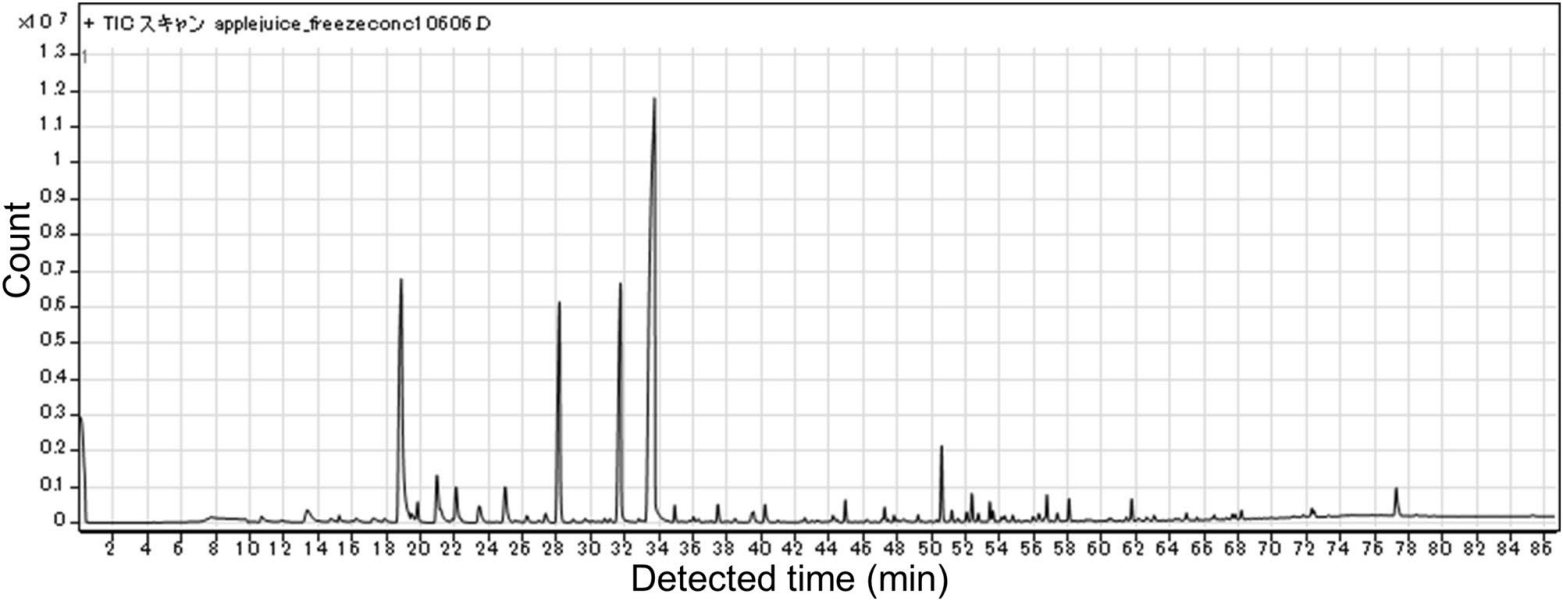
**C**hromatogram of apple juice concentrated by freezing.

We found that the number of generated compounds following freeze concentration of apple juice was larger than that after heat concentration (Fig. 4). The total number of compounds in fresh juice (97) and freeze-concentrated juice (57 original + 37 newly generated = 94) were similar. Individual chromatograms of each sample (fresh juice, concentrated by heating, and concentrated by freezing) are shown in Figs. 5, 6, and 7. We extracted 13 flavors for comparison based on previous studies^2,28^. Flavors in each detected sample are summarized in Table 1. Miyawaki et al. reported that 2-methyl-1-butanol, hexyl acetate, and hexanol are retained after fermentation of apple juice^2^. In the present study, 2-methyl-1-butanol was detected in fresh juice and freeze-concentrated juice, whereas hexyl acetate and hexanol were detected in all samples, including heat-concentrated samples. They also reported that isoamyl alcohol and phenetyl alcohol were detected only in fermented samples as fermentation-specific flavors. However, in our study, isoamyl alcohol was detected in fresh juice and phenetyl alcohol was detected in both fresh and freeze-concentrated juice. It is possible that apple juice already contains these flavors without fermentation and our study improved the detection ability over headspace-GC. A previous study reported that high sugar concentration allowed better detection^30^. However, we did not use the headspace method for detection, although SBSE detection might have had similar effects. Moreover, slight fermentation might have occurred due to contamination by yeast or microorganisms at any stage of the present study. We detected butanoic acid, sec-butyl acetate, and nonanal only in freeze-concentrated juice. Although butanoic acid produces a foul odor for quality purposes, sec-butyl acetate and nonanal are sweet flavors. Nonanal is a flavor found in peanuts and is reported to attract mosquitoes^32^. Our present study using SBSE methods detected many compounds including flavors. Therefore, the SBSE method may have the advantage of detecting flavor compounds more precisely. We demonstrated that freeze concentration retains more original flavors compared with heat concentration.

**Table 1:** Comparison of flavor detection in intact juice (Juice), heated concentrate (Heating), and frozen concentrate (Frozen).

No	Component	Juice	Heating	Frozen
1	Acetic acid	D^a^	D	D
2	Butanoic acid	ND^b^	ND	D
3	Butyl acetate	D	D	D
4	*Sec*-butyl acetate	ND	ND	D
5	Decanoic acid	D	ND	D
6	Ethyl hexanoate	D	D	D
7	Hexanol	D	D	D
8	Hexyl acetate	D	D	D
9	Isoamyl alchohol	D	ND	ND
10	2-Methylbutyl acetate	D	ND	D
11	Nonanal	ND	ND	D
12	Octanoic acid	D	ND	D
13	Phenetyl acid	D	ND	D

^a^Detected.

^b^Not detected.

### Recommendation for freeze-concentrated apple juice

**Figure 8:**
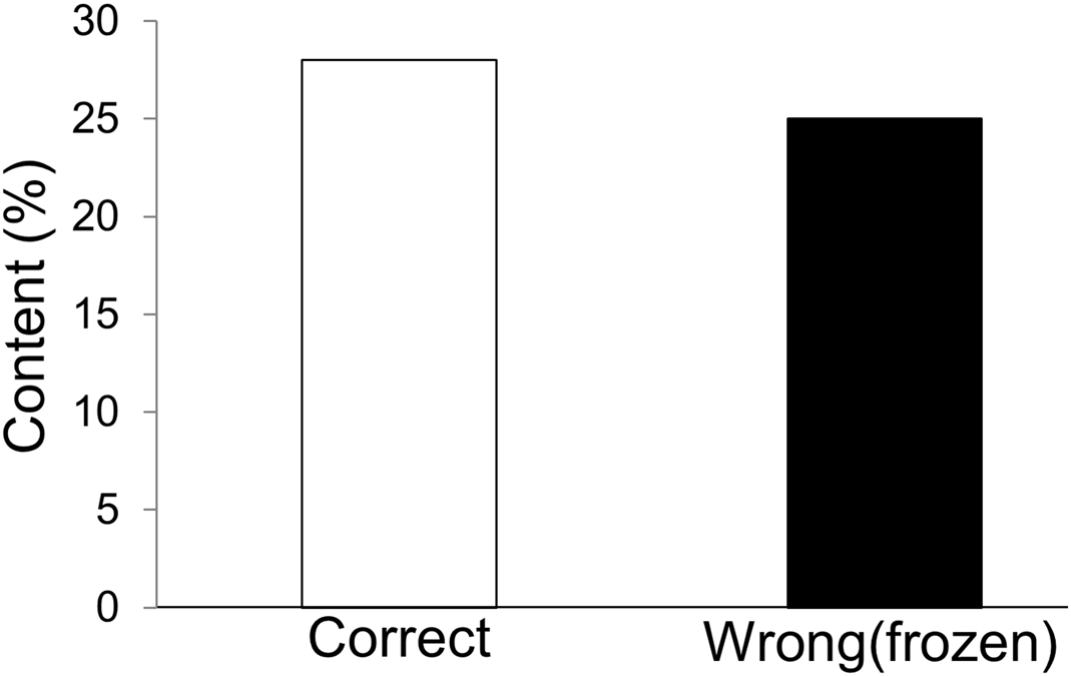
Results of tasting examination. Content ratio of total correct answers (white) and incorrect answers (wrong) where subjects confused frozen and heat-concentrated juice (black).

Although we understand that sensory evaluations are usually performed by trained panelists, in this study, we were interested in learning the reactions of general consumers because they have the largest influence on supermarket sales. The results of the triangle test from volunteers who were asked to blindly distinguish between fresh, heat-concentrated, and freeze-concentrated juice by taste are shown in Fig. 8. Interestingly, the number of people who correctly identified (16 people, 28%) and those who incorrectly identified fresh and reconstituted freeze-concentrated juice (14 people, 25%) was similar. When one answer is selected with a probability of 1/6, and more than 14 out of 57 people have a probability of 5% or less, the results are statistically significant. When volunteers were asked to choose only between fresh and reconstituted frozen juice (two-point test), 20 of 32 people correctly identified. As when one answer is selected with a probability of 1/3, more than 15 out of 32 people have a probability of 5% or less, these results are statistically significant. Therefore, we speculated that some people had difficulty identifying fresh juice from frozen concentrated juice. However, after the experiment, some people stated that the freeze-concentrated juice had a smell that was different to fresh juice. This may be the butanoic acid because this flavor was detected only in freeze-concentrated juice (Table 1). Results are summarized in Table 2 based on age and sex for each respondent. Although age and sex of a respondent may affect the answer, this was not obvious because the number of participants in each category was small. In the tasting test, some people had difficulty differentiating between fresh and frozen juice. In a previous study, sensory evaluations were performed by trained panelists who measured odor, aroma, flavor, and global assessments for both fresh and freezing concentration apple juice^18^. There were no significant differences for any of the abovementioned measures^18^. These results are in line with our study results, although our study investigated not for identify view point or judgment by untrained people. A similar study for flavor detection and human sensory evaluation was also conducted for both fresh and freezing concentrated calafate juice^17^. We also investigated flavor characteristics of freezing concentrated juice.

**Table 2:** Results of drink detection.

Years old	Less than 10		10–19		21–29		30–39		40–49		50–59		Over 60		Total
Sex	Men	Women	Men	Women	Men	Women	Men	Women	Men	Women	Men	Women	Men	Women	
**A**															
Correct	2	2	1	3			2	3	1	1			1		16
Wrong (inact and frozen		2		3				1		4	2			2	14
Others	1	2	2	4		1		2	2	3	2	3	3	2	17
Total	3	6	3	10	0	1	2	6	3	8	4	3	4	4	57
**B**															
Correct	3	2		2	0	0	0	2	2	1	4	2	1	1	20
Wrong	1	1		0	0	0	0	1	0	1	6	0	1	1	12
Total	4	3	0	2	0	0	0	3	2	2	10	2	2	2	32

Each answer is shown based on age and sex. A shows the results of three types of juice judgments, and B shows the results of two types of juice judgments.

Previously, it was reported that the freeze-concentrated juice and original juice did not differ in flavor^10^. Recently generated flavors are detected in freeze-concentrated juice^18,28^. Our study findings agree with those of these previous studies. Another study has indicated that one of the mechanisms for the detection of flavors only freezing concentrations, cause to detect easily at headspace by high sugar contents at head space detection^30^. Although SBSE methods have high sensitivity for flavors influenced by increased sugar content, there may be other mechanisms involved in the freezing process.

Similar phenomena have been reported whereby the effective material ornithine and its precursor, acorbine, from the bivalve *Corbicula japonica*^33,34^ and gamma-aminobutyric acid from the Chinese yam^33^ are increased during controlled freezing processes. Proposed mechanisms of such phenomena include increased enzyme activity in low-temperature environments^35^. In our case, newly generated flavors were detected in freeze-concentrated juice. Although we do not yet currently understand the mechanisms, we consider this an interesting phenomenon. We speculate that the relatively large increase in enzymatic activity or other mechanisms cause flavor generation, and freezing concentration plays a major role in this process. We are currently investigating this interesting phenomenon of flavor generation during freeze-concentration and plan to report findings in the near future.

Concentrated juice will be used as a source of other types of foods products such as alcoholic beverages. Food products with rich aromatic flavors generally have high quality. Therefore, freeze-concentrated juice should be of higher quality than heat-concentrated juice. Indeed, a large Japanese company has also applied the method of frozen concentration for grape juice^36^.

Our methods could make the concentration of juice simply to freeze and to melt. The apple production area in Japan (e.g., the Aomori prefecture) has very cold winters, often below 0 °C. In such environments, juice is placed outside to freeze naturally and the melting process only requires room temperature. Other methods of membrane concentration require extra equipment, such as membranes, which must be changed or washed^6^. The freeze concentration process is natural and does not require these extra costs.

## Conclusion

We successfully concentrated juice by heating and freezing. GC/MS analysis revealed that freeze-concentrating juice retains more than half of the compounds of fresh juice. Flavor retention achieved by freezing may be applicable for production of other foods. Furthermore, people had difficultly differentiating intact juice from frozen concentrated juice. Sensory examinations revealed that the ratios of those who correctly identified and those who incorrectly identified fresh and reconstituted freeze-concentrated juice were similar. We also discussed the mechanisms of flavor generation on freezing concentration with reference to the relatively large increase in enzymatic activity. Our study demonstrated that methods of juice concentration that utilize freezing retain flavor better. Therefore, freezing concentration methods are likely to be useful for numerous applications. Our study findings may have future benefits for freeze-concentrating apple juice as well other fruit juices.

## Data Availability

Research data have been provided in the manuscript.
